# Triage of hrHPV-positive women: comparison of two commercial methylation-specific PCR assays

**DOI:** 10.1186/s13148-020-00963-w

**Published:** 2020-11-11

**Authors:** Carolin Dippmann, Martina Schmitz, Kristina Wunsch, Stefanie Schütze, Katrin Beer, Christiane Greinke, Hans Ikenberg, Heike Hoyer, Ingo B. Runnebaum, Alfred Hansel, Matthias Dürst

**Affiliations:** 1 Klinik und Poliklinik für Frauenheilkunde und Fortpflanzungsmedizin, Universitätsklinikum Jena, Am Klinikum 1, 07747 Jena, Germany; 2 Oncgnostics GmbH, Winzerlaer Straße 2, 07745 Jena, Germany; 3 MVZ CytoMol, Berner Straße 76, 60437 Frankfurt am Main, Germany; 4 Institut Für Medizinische Statistik, Informatik und Datenwissenschaften, Universitätsklinikum Jena, Bachstraße 18, 07743 Jena, Germany

**Keywords:** Cervical cancer, CIN, DNA methylation, HPV, Triage, Epigenetic markers

## Abstract

**Aim:**

High-risk human papillomavirus (hrHPV)-based screening is becoming increasingly important, either by supplementing or replacing the traditional cytology-based cervical Pap smear. However, hrHPV screening lacks specificity, because it cannot differentiate between transient virus infection and clinically relevant hrHPV-induced disease. Therefore, reliable triage methods are needed for the identification of HPV-positive women with cervical intraepithelial neoplasia (CIN) in need of treatment. Promising tools discussed for the triage of these patients are molecular diagnostic tests based on epigenetic markers. Here, we compare the performance of two commercially available DNA methylation-based diagnostic assays—GynTect® and the QIAsure Methylation Test—in physician-taken cervical scrapes from 195 subjects.

**Findings:**

Both GynTect® and the QIAsure Methylation Test detected all cervical carcinoma and carcinoma in situ (CIS). The differences observed in the detection rates between both assays for the different grades of cervical lesions (QIAsure Methylation Test: CIN1 26.7%, CIN2 27.8% and CIN3 74.3%; GynTect®: CIN1 13.3%, CIN2 33.3% and CIN3 60%) were not significant. Concerning the false-positive rates, significant differences were evident. For the healthy (NILM) hrHPV-positive group, the false-positive rates were 5.7% for GynTect® and 26.4% for QIAsure Methylation Test (*p* = 0.003) and for the NILM hrHPV-negative group 2.2% vs. 23.9% (*p* = 0.006), respectively. When considering hrHPV-positive samples only for comparison (*n* = 149), GynTect® delivered significantly higher specificity compared to the QIAsure Methylation Test for CIN2 + (87.6% vs. 67.4% (*p* < 0.001)) and CIN3 + (84.1% vs. 68.2% (*p* = 0.002)).

Overall our findings suggest that DNA methylation-based tests are suitable for the triage of hrHPV-positive women. With the goal to provide a triage test that complements the limited specificity of HPV testing in HPV-based screening, GynTect® may be preferable, due to its higher specificity for CIN2+ or CIN3+ .

## Background

Cervical cancer (CxCa) is one of the most common cancers among women worldwide with both incidence and mortality rising: 570,000 newly diagnosed cases and 311,000 deaths in the year 2018 [1].

Through the introduction of screening programs, the incidence and mortality of cervical cancer decreased in many European countries, Australia/New Zealand and North America [1, 2]. Cytology-based diagnostics—the so-called Pap test—is the most widely used cervical cancer screening method. However, this method shows limited sensitivity for precancerous lesions [3].

For the development of cervical precancerous lesions and cancer, a persistent infection with high-risk human papillomaviruses (hrHPV) has been recognized as a necessary cause [4, 5]. hrHPV DNA can be identified in up to 99.7% of cervical cancers worldwide [4]. Based on these facts, testing for hrHPV infection is a highly sensitive tool for cervical cancer screening. HPV-based screening overcomes the limited sensitivity of cytology regarding precancerous lesions [3]. HPV-based screening, however, lacks specificity, and it is not possible to distinguish transient from persistent, clinically relevant hrHPV infections [6]. Furthermore, among young women HPV prevalence is high with up to 50% of the women aged below 29 years in the USA being infected with hrHPV [7]. Therefore, reliable triage methods are needed for hrHPV-positive women to identify those with cervical intraepithelial neoplasia (CIN) in need of treatment.

Different triage methods are discussed, and these include hrHPV genotyping, p16/Ki-67 cytological dual staining, altered expression profile of viral genes, and viral or host cell DNA methylation analysis [8, 9]. A promising tool, which gained most attention recently for the triage of hrHPV-positive women, is the application of host cell DNA methylation marker analysis [10]. The change of DNA methylation patterns—especially hypermethylation of promoter and 5′ regions of tumour suppressor genes—is an early event in carcinogenesis [11, 12] and may thus be very useful in cancer diagnostics [13].

In the field of colorectal cancer diagnosis, Cologuard® and Epi proColon® have played a pioneering role. Both assays are based on the detection of the hypermethylation either of the genes *NDRG4* and *BMP3* (Cologuard®) or *SEPT9* (Epi proColon®) [14, 15]. A methylation analysis of the biomarker *SEPT9* is also used for the detection of hepatocellular carcinoma (HCCBloodTest) [16]. For cervical cancer screening triage, the two best known commercially available DNA methylation-based diagnostic assays are: (1) GynTect®, based on methylated DNA regions in the promoter/5′ regions of the genes *ASTN1, DLX1, ITGA4, RXFP3, SOX17, and ZNF671* and (2) the QIAsure Methylation Test with the associated methylation markers *FAM19A4* and *miR124-2*.

In the present work, we investigated the performance of GynTect® in comparison with the QIAsure Methylation Test as triage assays in 195 cervical scrapes.

## Results

In this study, the DNA methylation-based diagnostic assays GynTect® and the QIAsure Methylation Test were assessed for their performance as triage tests for hrHPV-positive women. For this comparison, 195 cervical scrapes were analysed by both assays. Of these, 46 samples were cytologically normal (NILM) and hrHPV-negative, and all other samples were hrHPV-positive. The mean age of the patients was 40.5 years (range 19–84).

Overall, the QIAsure Methylation Test had a somewhat higher detection rate among CIN samples than GynTect®, but these differences were not significant. In contrast, significant differences in the detection rates were observed for NILM samples, irrespective of the hrHPV status (Table 1).

**Table 1:** Detection rates of both assays according to histological/cytological and hrHPV findings (*n* = 195)

	GynTect®		QIAsure Methylation Test		*P* value for comparison of detection rate
	Test positive	Detection rate [%] (95% CI)	Test positive	Detection rate [%] (95% CI)	
hrHPV +					
CxCa (*n* = 2)	2	2/2*	2	2/2*	–
CIS (*n* = 5)	5	5/5*	5	5/5*	–
CIN3 (*n* = 35)	21	60.0 (42.1–76.1)	26	74.3 (56.7–87.5)	0.125
CIN2 (*n* = 18)	6	33.3 (13.3–59.0)	5	27.8 (9.7–53.5)	1.000
CIN1 (*n* = 15)	2	13.3 (1.7–40.5)	4	26.7 (7.8–55.1)	0.500
no CIN (*n* = 21)	6	28.6 (11.3–52.2)	11	52.4 (29.8–74.3)	0.125
NILM (*n* = 53)	3	5.7 (1.2–15.7)	14	26.4 (15.3–40.3)	0.003
hrHPV −					
NILM (*n* = 46)	1	2.2 (0.1–11.5)	11	23.9 (12.6–38.8)	0.006

*Due to the low number of cases we present absolute frequencies for description and omit percentages and statistical comparisons

In Table 2, the diagnostic performance of the GynTect® assay and QIAsure Methylation Test with respect to CIN2+ and CIN3+ in terms of sensitivity and specificity is summarized. Importantly, these measures were estimated for the hrHPV-positive subgroup only, since the tests were designed as triage option for this patient group. Significant differences are only evident for specificity in case of CIN2+ and CIN3+ (Table 2).

**Table 2:** Clinical performance of both assays regarding the detection of CIN2+ and CIN3+ in the hrHPV-positive subgroup (*n* = 149)

	True positive	Sensitivity [%] (95% CI)	True negative	Specificity [%] (95% CI)
CIN2 +				
GynTect®	34/60	56.7 (43.2–69.4)	78/89	87.6 (79.0–93.7)
QIAsure Methylation Test	38/60	63.3 (49.9–75.4)	60/89	67.4 (56.7–77.0)
*P* value		0.424		< 0.001
CIN3 +				
GynTect®	28/42	66.7 (50.5–80.4)	90/107	84.1 (75.8–90.5)
QIAsure Methylation Test	33/42	78.6 (63.2–89.7)	73/107	68.2 (58.5–76.9)
*P *value		0.125		0.002

Moreover, we calculated the predictive values for both assays for CIN3+ by applying the Bayes theorem for a meaningful disease prevalence. We assume a CIN3+ prevalence of 30% for a triage setting of hrHPV-positives and values for sensitivity and specificity as shown in Table 2. With this constellation, the positive predictive values would be 64.3% for GynTect® and 51.4% for the QIAsure Methylation Test and the negative predictive values would be 85.5% and 88.1%, respectively.

## Discussion

The limited sensitivity of cytology-based screening [3] has led to the introduction of HPV-based screening in several countries (e.g. Great Britain, Netherlands, San Marino, Turkey, Germany) [17, 18]. The specificity of HPV testing for cervical cancer, however, is very low. Recently, Leeman et al. reported a high sensitivity of hrHPV testing for CIN3+ of 90.3%, but a specificity of only 31.8% [19]. These findings are consistent with many previous studies [20–22].

Hypermethylation in certain promoter regions is an early event in carcinogenesis. Thus, methylation markers are getting increasing awareness as promising triage tools for hrHPV-positive women [9, 11, 19, 23, 24], and as a consequence diagnostic tests are emerging based on this marker class [13].

With this study, we provide a comparison of the performance of two commercially available DNA methylation-based diagnostic assays—GynTect® and QIAsure Methylation Test—regarding sensitivity and specificity in a sample comprising 195 cervical scrapes. The purpose was to evaluate the assays as potential triage tools for HPV-based cervical cancer screening.

Our study confirmed the extremely high sensitivity of the DNA methylation tests for the detection of cancer cases. Both assays recognized all cancer and CIS cases. Similar results were also shown before for GynTect® with 123/123 CxCa detected and the QIAsure Methylation Test with 510/519 CxCa detected [9, 25]. Moreover, for GynTect® the detection rates for different stages of CIN ranged from 13.3% for CIN1 over 33.3% for CIN2 to 60% for CIN3. In a previous study, Schmitz et al. achieved detection rates within the same range (CIN1: 20%; CIN2: 44.4%; CIN3: 61.2%) [26]. For the QIAsure Methylation Test, the detection rates were 26.7% for CIN1, 27.8% for CIN2, and 74.3% for CIN3 (Table 1). With exception of the CIN2 cases, these positivity rates also correlate with previous studies (CIN1: 27.7%; CIN2: 44.3%; CIN3: 75.8%) [19].

Surgical removal of a precancerous lesion is the recommended treatment for histologically confirmed CIN2-3 [27]. Based on this recommendation, a more detailed assessment of the detection rates of CIN2 and CIN3 by the two assays is warranted. GynTect® detected around 33–60% of these lesions, whereas QIAsure Methylation Test showed detection rates of 28–74%. In this context, it is important to consider that not all CIN3 progress to cervical cancer [28] and that the majority of CIN1 and CIN2 regress without treatment. Wang et al. showed that among women with CIN2 (*n* = 25) 56% regressed to normal, 24% to CIN1, 4% remained as CIN2, and 16% progressed to CIN3+ within six years [29]. Loopik et al. reported similar data—regression of CIN2 in 71.1% of cases and a progression from CIN2 to CIN3 in 16.6% of cases among women younger than 25 (*n* = 150) [30]. In the light of these studies, it is clear that an ideal triage test should allow to discriminate between high-grade lesions with a low risk for progression and clinically relevant lesions, which obviously are not distinguishable by histopathology. The two assays investigated in the present study may provide such a possibility. However, this needs to be confirmed in prospective observational studies which are currently ongoing.

Regarding the detection rates within the “no CIN” group (GynTect®: 28.6%, QIAsure Methylation Test: 52.4%), it is important to note that this population comprises exclusively women who were referred to colposcopy for diagnostic workup. These patients showed abnormal cytology and hrHPV positivity. However, the biopsies taken revealed no CIN. In a recently published study, the positivity rate of the QIAsure Methylation Test was 23.2% for hrHPV-positive “no CIN” cases and 25.4% for CIN1 [31]. We have no explanation for the high QIAsure positivity rate for “no CIN” samples in our study population. Nevertheless, the methylation rate among “no CIN” samples is markedly higher than for CIN1 in the respective assays, suggesting that in some cases biopsy may have missed subclinical high-grade lesions.

Of particular interest is the performance of the assays for hrHPV-negative NILM cases. In this group, 2.2% were positive for GynTect®, but 23.9% for the QIAsure Methylation Test (*p* = 0.006). Considering the fact that a persistent infection with hrHPV types is a prerequisite for cervical carcinogenesis, the methylation rate among hrHPV-negative NILM cases reflects unspecific background methylation. We could not find any data in the literature reporting results of the QIAsure Methylation Test for hrHPV-negative NILM cases only. Also for hrHPV-positive NILM cases, the QIAsure Methylation Test has much higher positivity rates compared to GynTect® (26.4% vs 5.7%, respectively, *p* = 0.003). As for the hrHPV-negative NILM cases, we could not find published data on the performance of the QIAsure Methylation Test for hrHPV-positive NILM cases only. Thus, in a triage setting, it is very likely that the QIAsure Methylation Test would lead to higher colposcopy referrals in comparison with the GynTect® assay.

Besides the methylation markers *ASTN1, DLX1, ITGA4, RXFP3, SOX17, ZNF671* (GynTect®) and *FAM19A4, miR124-2* (QIAsure Methylation Test), other markers are discussed [23, 24, 32]. Bierkens et al. showed an increased methylation level of the markers *CADM1* and *MAL* with increasing severity of the lesion (5.3–6.2-fold in CIN2/3 and 143.5–454.9-fold in cervical cancer cases) [23]. In another study, *CADM1* and *MAL* displayed a CIN3+ sensitivity of 70% and a CIN3+ specificity of 78% in the triage of hrHPV-positive women [32]. A four-gene methylation marker panel consisting of the markers *JAM3, EPB41L3, TERT* and *C13ORF18* revealed a CIN3+ sensitivity of 84% and a specificity of 69% for hrHPV-positive cervical scrapes [24]. Compared to the results achieved in the current study, the sensitivity of the above-mentioned methylation markers is in a similar range. Regarding specificity, however, GynTect® shows the best results for CIN3+ .

## Conclusion

An ideal screening strategy for cervical cancer is characterized by maximum sensitivity to detect all cases with clinically relevant disease and maximum specificity to reduce false positive results and ultimately also overtreatment. Methylation-based triage tests for hrHPV-positive women are highly promising. In particular, the GynTect® assay convinces with high sensitivity and unmatched specificity.

## Methods

### Patient samples

Cervical scrapes were collected from patients attending the dysplasia unit at the Department of Gynaecology and Reproductive Medicine at the Jena University Hospital (Germany). Further samples were available from a previous study conducted with CytoMol, Frankfurt, Germany. All of the above samples comprise cervical scrapes in PreservCyt® liquid-based cytology (LBC) media (Hologic, Wiesbaden, Germany).

For each sample, information regarding cytology or, in cases in which a biopsy was taken, the histopathological diagnosis was available. The samples included in this study had the following cytology findings or histopathological diagnosis: normal cytology (NILM) and hrHPV-negative (*n* = *46*); NILM and hrHPV-positive but no biopsy taken (*n* = *53)*; colposcopically suspect but normal histopathology on biopsy (= no CIN) (*n* = *21*); CIN1 (*n* = *15*); CIN2 (*n* = *18*); CIN3 (*n* = *35*); CIS (*n* = *5*) and CxCa (*n* = *2*). All cases of no CIN, CIN1, CIN2, CIN3, CIS, and cancers were hrHPV-positive.

#### DNA isolation and HPV testing

For the QIAsure Methylation Test (Qiagen, Hilden, Germany), DNA was isolated from 5 ml of the LBC sample by use of the NucleoSpin® Tissue Kit (Macherey Nagel, Düren, Germany) according to the manufacturer’s instructions. Concentration of genomic DNA was measured using a NanoDrop 2000 UV–Vis spectrophotometer (VWR, Erlangen, Germany). HPV status of the samples was determined using the GP5+ /6+ PCR-EIA assay [33] or the cobas® HPV test (Roche, Mannheim, Germany).

#### DNA methylation marker analysis

##### GynTect®

The GynTect® assay (oncgnostics GmbH, Jena, Germany), which analyses the six DNA methylation markers *ASTN1, DLX1, ITGA4, RXFP3, SOX17, ZNF671* and two controls (*ACHE, IDS*), was performed for all samples as described in the instructions for use (oncgnostics GmbH, Jena, Germany).

Briefly, LBC samples were vortexed and 1 ml was immediately transferred into a 2-ml reaction tube. Cells were pelleted by centrifugation, and 900 µl supernatant was discarded. 40 µl of the resuspended cells was used for bisulfite treatment using the EpiTect® Fast Bisulfite Kit (Qiagen, Hilden, Germany) following the supplier’s manual without previous DNA isolation. After elution (in 20 µl), the sample volume was increased by adding 70 µl water.

The GynTect® methylation-specific real-time PCR was performed using custom-made real-time PCR Master Mix (MM), containing a Hotstart DNA polymerase. 10 µl of this MM was added to each vial in an eight-tube strip, each containing a different pair of the pre-dried primers for the respective markers. 10 µl of the bisulfite-converted DNA, serving as a template for each marker, was added to each tube. The PCRs were performed using the ABI7500 PCR system (Life Technologies, Thermo Fisher Scientific, USA). A detailed description of the GynTect® methylation-specific real-time PCR has been published previously [21].

For each methylation marker, the Ct value was determined and a delta Ct was calculated using the Ct value of the methylation control marker *IDS* as reference (prerequisite: Ct value IDS ≤ 32). To score a methylation marker as positive, the delta Ct (Ct_Marker_ – Ct_IDS_) has to be ≤ 8 for *ASTN1*, ≤ 9 for *DLX1*, *ITGA4, RXFP3*, and *SOX17*, and ≤ 10 for *ZNF671*. The whole GynTect® assay was considered positive if the amount of all methylation marker scores was 6 or higher (single-marker scores *DLX1*: 1; *ASTN1, ITGA4, RXFP3, SOX17*: each 2; and *ZNF671*: 6).

##### QIAsure Methylation Test

The QIAsure Methylation Test (Qiagen, Hilden, Germany) analyses in a multiplex methylation-specific real-time PCR the methylation of the promoter regions of *FAM19A4* and *miR124-2* and a reference gene (*ACTB*). The test was performed for all samples as described in the instructions for use (Qiagen, Hilden, Germany).

Briefly, genomic DNA was isolated and its concentration measured as described in “DNA isolation and HPV testing” above. In the bisulfite reaction up to 300 ng/45 µl, isolated genomic DNA was converted using the EZ DNA Methylation Kit following the instructions of the supplier (Zymo Research Europe, Freiburg, Germany). DNA was eluted in 10 µl.

To perform the QIAsure Methylation Test, 17.5 µl custom-made real-time PCR MM and 2,5 µl template were needed. The multiplex PCR was run on a Rotor-Gene® Q MDx 5plex system (Qiagen, Hilden, Germany).

The samples were scored hypermethylation-positive if the Ct value of *ACTB* was ≤ 26.4, and at least one of the methylation marker genes had a ΔΔCt below the cut-off [19].

#### Statistical evaluation

For both assays, detection rate was calculated according to the histologically confirmed cervical disease status (cytology for the NILM group). Sensitivity and specificity were estimated for the diagnosis of CIN2 or higher (CIN2+) and CIN3 or higher (CIN3+) with exact two-sided 95% confidence intervals (CI) assuming a binomial distribution. Performance of both methylation-based diagnostic assays was statistically compared by the McNemar’s test. The two-sided level of significance was set to 0.05. Predictive values were calculated for a reasonable prevalence by applying the Bayes theorem. Statistical analysis was performed using GraphPad Prism (Version 5.00 for Windows, GraphPad Software, San Diego, California, USA) and SAS (Version 9.4).

## Data Availability

The raw data generated in this study are available from the corresponding author on reasonable request.

## Abbreviations

ACHE: Acetylcholinesterase
ACTB: β-Actin
ASTN1: Astrotactin 1
BMP3: Bone morphogenetic protein 3
C13ORF18: Rubicon-like autophagy enhancer
CADM1: Cell adhesion molecule 1
CI: Confidence interval
CIN: Cervical intraepithelial neoplasia
CIS: Carcinoma in situ
Ct: Cycle threshold
CxCa: Cervical cancer
DLX1: Distal-less homeobox 1
DNA: Deoxyribonucleic acid
EPB41L3: Erythrocyte membrane protein band 4.1 like 3
FAM19A4: Family with sequence similarity 19 member A4
HPV: Human papillomavirus
hrHPV: High-risk HPV
IDS: Iduronate 2-sulfatase
ITGA4: Integrin subunit alpha 4
JAM3: Junction adhesion molecule 3
Ki-67: Marker of proliferation
LBC: Liquid-based cytology
MAL: T-lymphocyte maturation-associated protein
miR124-2: MicroRNA 124
MM: Master mix
NDRG4: N-Myc downstream-regulated gene 4
NILM: Negative for intraepithelial lesion and malignancy, meaning normal cytology
p16: Cyclin-dependent kinase inhibitor 2A
PCR: Polymerase chain reaction
RXFP3: Relaxin/insulin-like family peptide receptor 3
SEPT9: Septin 9
SOX17: Sex determining region Y-box 17
TERT: Telomerase reverse transcriptase
ZNF671: Zinc finger protein 671
